# Primary rectal mucosa-associated lymphoid tissue lymphoma in a patient with previously identified primary biliary cirrhosis and secondary Sjögren’s syndrome

**DOI:** 10.1007/s12328-016-0643-x

**Published:** 2016-04-01

**Authors:** Kazumasa Kawashima, Kyoko Katakura, Yuta Takahashi, Hiroyuki Asama, Tatsuo Fujiwara, Hiromi Kumakawa, Hiromasa Ohira

**Affiliations:** Department of Gastroenterology, Public Soma General Hospital, Fukushima, Japan; Department of Gastroenterology and Rheumatology, Fukushima Medical University, 1 Hikarigaoka, Fukushima, 960-1295 Japan

**Keywords:** Primary biliary cirrhosis, Rectal MALT lymphoma, Secondary Sjögren’s syndrome

## Abstract

An 83-year-old female began treatment with prednisolone and ursodeoxycholic acid at 62 years of age, following a diagnosis of primary biliary cirrhosis (PBC) and secondary Sjögren’s syndrome (SjS). With persisting bloody stools, the patient underwent colonoscopy at 83 years of age. Histopathological evaluation revealed mucosa-associated lymphoid tissue (MALT) lymphoma. The elevated rectal lesion resolved with rituximab treatment. We report this case because although patients with SjS are at increased risk of malignant lymphoma, primary rectal MALT lymphoma is very uncommon in association with PBC and secondary SjS.

## Introduction

Optimal treatment for rectal mucosa-associated lymphoid tissue (MALT) lymphoma, an uncommon type of lymphoma, has yet to be established. Recently, several cases of complete response for MALT lymphoma have been achieved using rituximab monotherapy as an alternative to the conventional treatments of *Helicobacter pylori* eradication and surgery [1–4]. In addition, patients with Sjögren’s syndrome (SjS) show a 2.6-fold greater risk of developing a malignancy and a 37.5-fold greater risk of developing non-Hodgkin’s lymphoma relative to healthy individuals [5]. Primary biliary cirrhosis (PBC) is occasionally listed as a complication of SjS, but in our search of the relevant literature, MALT lymphoma as a complication of SjS and PBC was confined to bone marrow, lacrimal glands, lungs, and liver [6, 7]. These cases were treated with chemotherapy, radiation and surgery, respectively.

Along with a brief review of the literature, we report a rare case of primary rectal MALT lymphoma responding to rituximab monotherapy in a patient undergoing long-term follow-up for PBC and SjS.

## Case report

**Fig. 1:**
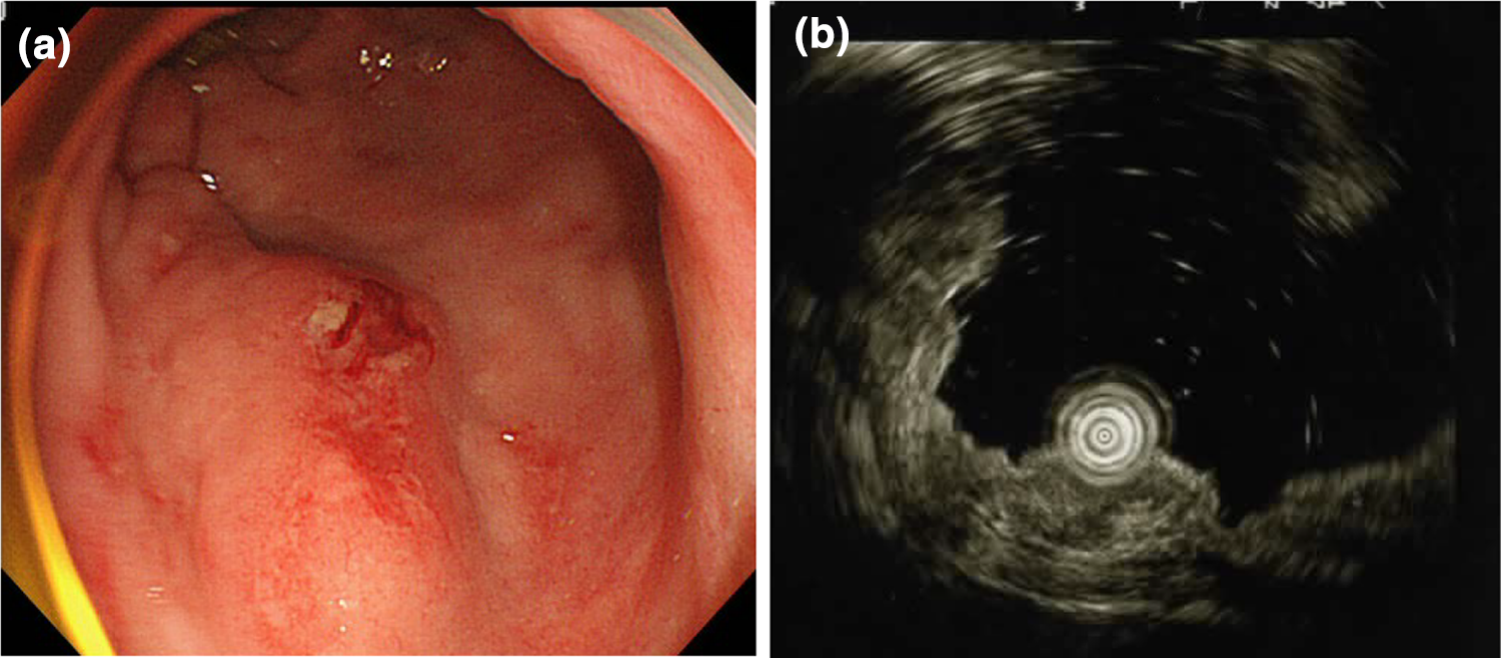
Images from proctoscopy and endoscopic ultrasonography. **a** An endoscopic image shows enlargement of the elevation and a reddish depression at the center of the lesion in the lower rectum (Rb). **b** Endoscopic ultrasonography shows the tumor to be situated in the second to third layers. Internal echo is non-uniform and slightly hypoechoic. No invasion into the fourth layer is evident

In 1993, a 62-year-old female was referred from her primary doctor for further evaluation for Raynaud’s symptoms and abnormal hepatic function. She showed positive results for anti-mitochondrial M2 antibody (AMA-M2) at 59.8 index, and although liver biopsy revealed no evidence of chronic non-suppurative destructive cholangitis, was diagnosed with PBC based on histological findings of bile duct destruction. At the same time the patient was diagnosed with PBC she showed negative results for anti-SS-A and anti-SS-B antibodies, despite complaining of thirst and eye dryness. She was examined at the Department of Otolaryngology at our hospital and was diagnosed with SjS secondary to PBC based on a positive Schirmer’s test result and labial gland biopsy results. Ursodeoxycholic acid treatment (600 mg/day) and prednisolone (PSL) treatment (30 mg/day) were initiated. PSL was subsequently tapered and discontinued in 2007. Ursodeoxycholic acid treatment was continued on an outpatient basis. The patient underwent lower gastrointestinal tract endoscopy in December 2013 for bloody stools. A mildly red elevated lesion measuring 5 mm maximum diameter was found in the rectum below the peritoneal reflection. An endoscopy performed in May 2014 showed the rectal lesion to have transformed to a pitted, red submucosal tumor (Fig. 1a). The lesion appeared as normal mucosa on narrow-band imaging colonoscopy. A hypoechoic lesion was seen in the second to third layers on endoscopic ultrasonography (Fig. 1b). As the histopathological examination revealed no evidence of malignancy and infiltration of lymphoid cells, an immunohistochemical examination was not performed. Although the histopathological examination revealed no fibromuscular obliteration, the patient was diagnosed with rectal mucosal prolapse syndrome based on endoscopic findings of similarity with lymphoma. With mild bloody stools persisting, the patient was admitted to our department in July 2014 for further evaluation and treatment.

Tests on admission revealed thrombocytopenia and anemia. Liver function was classified as Child-Pugh class B based on blood biochemistry tests. Positive serum antinuclear antibody findings (1:640 speckled pattern) and a high AMA-M2 (41.3 index) were not inconsistent with PBC.

Soluble interleukin-2 receptor was slightly elevated, but all other tumor markers were within the normal ranges. Negative results were obtained for both anti-*H. pylori* antibody and *H. pylori* antigen stool test.

**Fig. 2:**
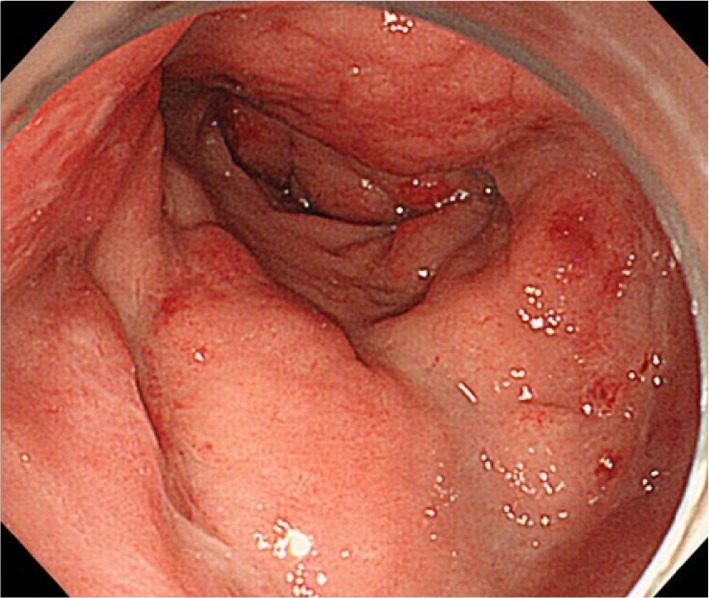
Colonoscopy performed on admission shows the rectal lesion to have become a hard elevated lesion measuring 30 mm maximum diameter. The surrounding mucosa is circumferentially red and edematous from Rb to Ra

**Fig. 3:**
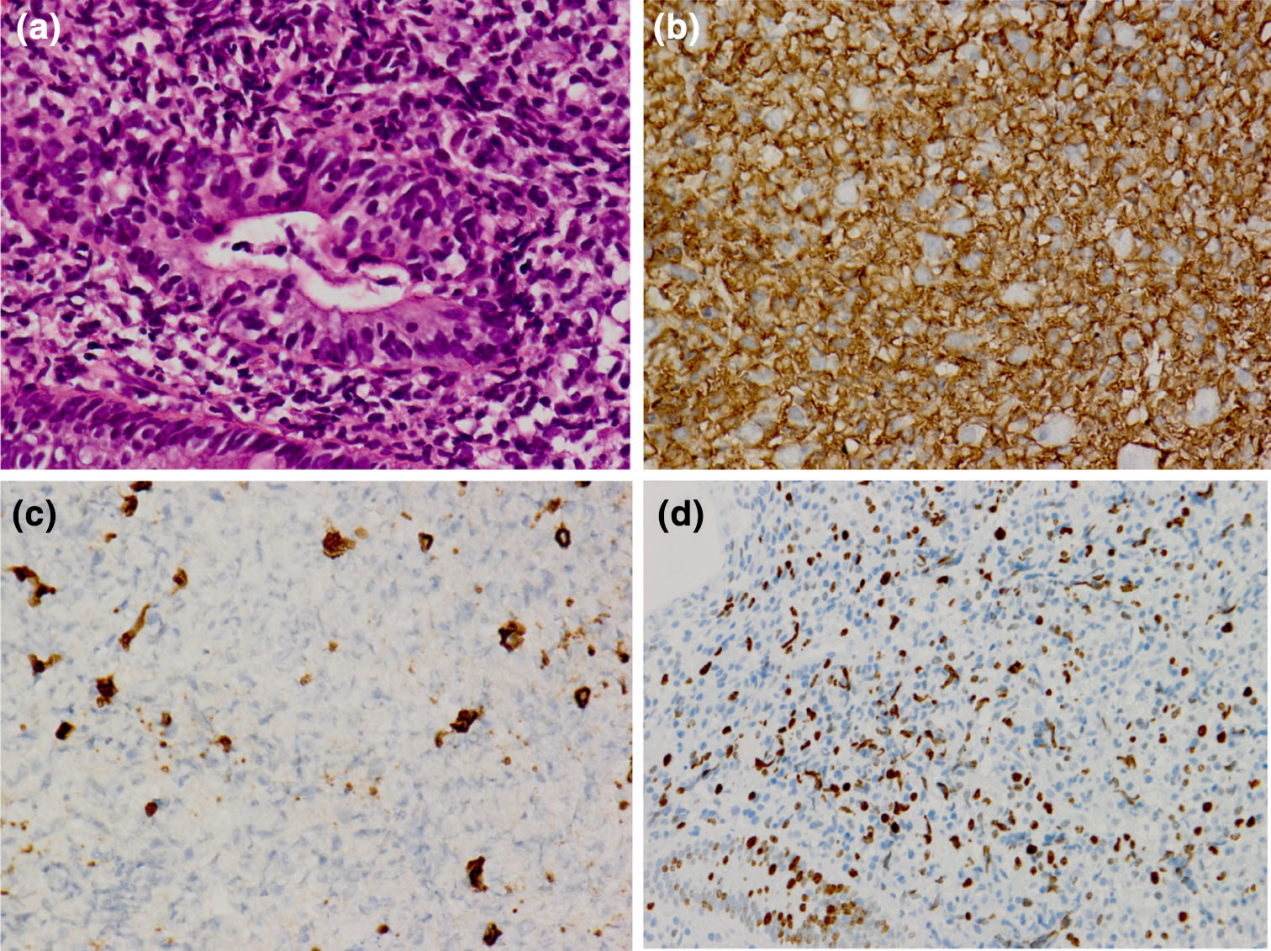
Histopathological examination of a colon biopsy sample shows infiltration of small to medium-sized lymphocytes. **a** H&E staining (magnification 400×) shows small to medium-sized atypical lymphocytes (centrocyte-like cells) densely infiltrating into the mucosa. Some lymphocytic infiltration is seen in the crypt epithelium (lymphoepithelial lesion). **b**, **c** Tissues show diffusely positive CD20 staining (**b**) and negative CD3 staining (**c**) (magnification 400×). **d** Immunohistochemistry of Ki-67 (MIB-1) shows the labeling index is 30 %

Colonoscopy performed in July 2014 showed the rectal lesion as a hard, reddish, elevated lesion measuring 30 mm maximum diameter (Fig. 2). Histopathological examination of the elevated lesion and surrounding mucosa with hematoxylin and eosin (H&E) staining showed dense infiltration of small to medium-sized lymphocytes into the mucosa, with some lymphocytic infiltration in the crypt epithelium (Fig. 3a). Tissues were diffusely positive for CD20 staining and negative for CD3 staining (Fig. 3b, c). κ light-chain staining was more pronounced than λ staining at the site. As Ki-67 (MIB-1) labeling index was 30 % and immunohistochemistry for CD5, cyclinD1, and CD10 showed negative staining, we diagnosed MALT lymphoma (Fig. 3d).

**Fig. 4:**
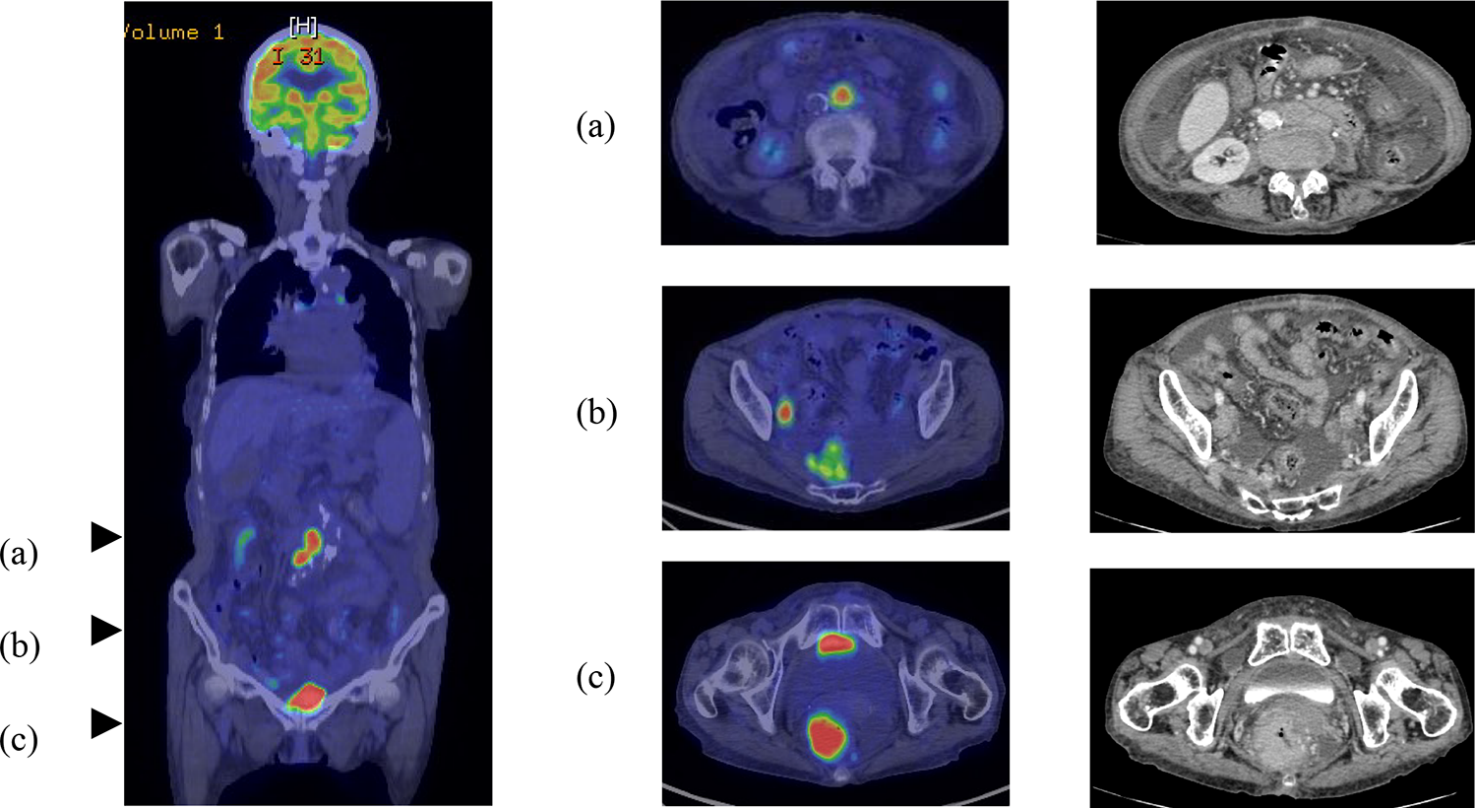
PET-CT and abdominal CT show a poorly demarcated high-absorption mass with surrounding inflammation measuring 65×42×70 mm in Rb (**c**). Accumulation into and enlargement of the para-aortic lymph nodes and lateral lymph nodes is apparent (**a**, **b**)

**Fig. 5:**
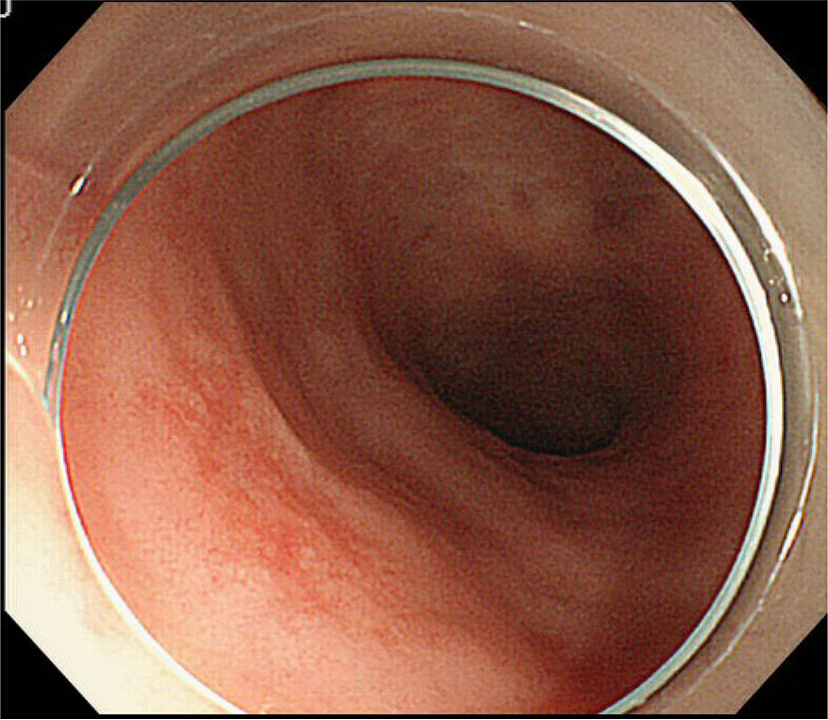
The lesion has flattened at 3 months post treatment

In contrast to abdominal computed tomography (CT) performed in December 2013, which showed only hepatic cirrhosis and splenomegaly, abdominal CT performed when the patient was admitted showed ascites, thickening of the rectal wall and evidence of surrounding inflammation, and enlargement of the lateral and para-aortic lymph nodes. Positron emission tomography/CT revealed circumferential fluorodeoxyglucose (FDG) accumulation in the rectum below the peritoneal reflection (Rb) and FDG accumulation in the lateral and para-aortic lymph nodes (Fig. 4). The lesion was classified using Lugano classification as stage II2 malignant lymphoma based on these findings. Duodenoscopy and CT revealed no other MALT lymphoma within the gastrointestinal tract. The patient refused aggressive examinations, such as enteroscopy and surgery because the hepatic cirrhosis was uncompensated, the prognosis for MALT lymphoma is relatively good, and surgery would have required colostomy. Although *H. pylori* eradication has proven effective in some patients showing negative results for the pathogen [8, 9], it was not performed for this patient because she had PBC and SjS, which indicated autoimmune involvement. Although upper gastrointestinal endoscopy showed slight mucosal atrophy in the antrum of the stomach, there was no *H. pylori* infection as well as no history of *H. pylori* eradication. Radiation therapy was effective for primary rectal MALT lymphoma with no distant spread [10–12]. In this case, four courses of once-weekly rituximab, which has a favorable safety profile, were prescribed at 375 mg/m^2^ with the consent of the patient and her family to treat persistent bleeding. A proctoscopy performed 3 months after treatment showed the elevated lesion to have flattened almost entirely (Fig. 5). A biopsy showed only mild lymphocytic infiltration. CT revealed reduction of rectal wall thickening and surrounding inflammation with no lymph node enlargement, as seen before rituximab treatment. The efficacy of rituximab monotherapy was partial remission of MALT lymphoma in this case. Further rituximab monotherapy was not performed for this patient because she had no bloody stools and did not wish further treatment.

## Discussion

Gastrointestinal lymphomas occur predominantly in the stomach (60–65 %) and small intestine (approximately 30 %). Lymphomas in the colon and rectum are rare (≤10 %) [13].

The patient, who was being followed for previously identified PBC and SjS, developed primary rectal MALT lymphoma with gastric mucosal atrophy on upper gastrointestinal endoscopy, but no *H. pylori* infection. SjS-associated malignant lymphoma is thought to result when chronic inflammation from autoimmunity causes T lymphocytes to release cytokines that cause B-lymphocyte proliferation and monoclonal transformation [14]. The resulting B-cell hyperplasia is thought to progress to MALT lymphoma over many years, and become highly malignant. Anywhere from 6 months to 29 years can separate SjS diagnosis from the onset of malignant lymphoma [15]. Immune reactions in glandular tissues cause SjS, while those in bile duct epithelial cells cause PBC [16]. Many years of lymphoid tissue immune reactions caused by PBC and SjS, in addition to infection, may therefore have been behind the pathogenesis of MALT lymphoma in this case [17].

In our search of the relevant literature, MALT lymphoma has been reported in patients with both PBC and SjS in only the liver, lungs, bone marrow, and lacrimal glands [6, 7]. These cases were treated with chemotherapy, radiation and surgery, respectively. Of 8 patients with primary hepatic MALT lymphoma described in a report by Kikuma et al., two had coexisting hepatitis C virus infection, and four had PBC, SjS, or autoimmune hepatitis [6]. Chronic inflammation caused by infection or persisting immune reactions appears to contribute to MALT lymphoma pathogenesis in patients with PBC, SjS, or other immunological disease.

Although MALT lymphomas can occur in patients without an autoimmune disease, we must consider the contribution not only of infection, but also of chronic inflammation in the body when managing rectal MALT lymphoma in patients with an autoimmune disease because the colon is home to countless microorganisms that constantly subject it to diverse antigenic stimulations and chemical attacks [18].

Rituximab has recently been used to treat MALT lymphoma resistant to eradication therapy and, as monotherapy, has shown a response rate of 40–70 %. The response rate of non-gastric MALT lymphomas to rituximab monotherapy is approximately 80 % [4]. The adverse effects associated with rituximab, such as nausea following the initial dose, are symptomatically treatable, making the drug relatively safe even in elderly patients. We selected rituximab monotherapy for our patient because she showed negative results for anti-*H. pylori* antibody and *H. pylori* antigen in the stools, had poor hepatic function due to PBC (Child-Pugh class B), and showed enlargement of the abdominal lymph nodes. The rectal lesion was markedly smaller after completing 4 courses and showed no evidence of recurrence at 3 months post treatment.

In conclusion, the relationship between MALT lymphoma of the rectum and PBC with SjS is not clear, although this case report indicates that MALT lymphoma can be caused by various factors associated with infections and immune reactions. Clinical results observed in our experience confirm that the use of rituximab monotherapy can be considered effective for inoperable patients with MALT lymphoma with autoimmune complications.
